# Characterization of *M. tuberculosis* SerB2, an Essential HAD-Family Phosphatase, Reveals Novel Properties

**DOI:** 10.1371/journal.pone.0115409

**Published:** 2014-12-18

**Authors:** Gaya Prasad Yadav, Sonal Shree, Ruchi Maurya, Niyati Rai, Diwakar Kumar Singh, Kishore Kumar Srivastava, Ravishankar Ramachandran

**Affiliations:** 1 Molecular and Structural Biology Division, CSIR-Central Drug Research Institute, Sector 10, Jankipuram Extension, Sitapur Road, Lucknow, Uttar Pradesh, 226031, India; 2 Microbiology Division, CSIR-Central Drug Research Institute, Sector 10, Jankipuram Extension, Sitapur Road, Lucknow, Uttar Pradesh, 226031, India; National Centre for Cell Science, India

## Abstract

*M. tuberculosis* harbors an essential phosphoserine phosphatase (MtSerB2, Rv3042c) that contains two small- molecule binding ACT-domains (Pfam 01842) at the N-terminus followed by the phosphoserine phosphatase (PSP) domain. We found that exogenously added MtSerB2 elicits microtubule rearrangements in THP-1 cells. Mutational analysis demonstrates that phosphatase activity is co-related to the elicited rearrangements, while addition of the ACT-domains alone elicits no rearrangements. The enzyme is dimeric, exhibits divalent metal- ion dependency, and is more specific for l- phosphoserine unlike other classical PSPases. Binding of a variety of amino acids to the ACT-domains influences MtSerB2 activity by either acting as activators/inhibitors/have no effects. Additionally, reduced activity of the PSP domain can be enhanced by equimolar addition of the ACT domains. Further, we identified that G18 and G108 of the respective ACT-domains are necessary for ligand-binding and their mutations to G18A and G108A abolish the binding of ligands like l- serine. A specific transition to higher order oligomers is observed upon the addition of l- serine at ∼0.8 molar ratio as supported by Isothermal calorimetry and Size exclusion chromatography experiments. Mutational analysis shows that the transition is dependent on binding of l- serine to the ACT-domains. Furthermore, the higher-order oligomeric form of MtSerB2 is inactive, suggesting that its formation is a mechanism for feedback control of enzyme activity. Inhibition studies involving over eight inhibitors, MtSerB2, and the PSP domain respectively, suggests that targeting the ACT-domains can be an effective strategy for the development of inhibitors.

## Introduction

**Figure 1:**
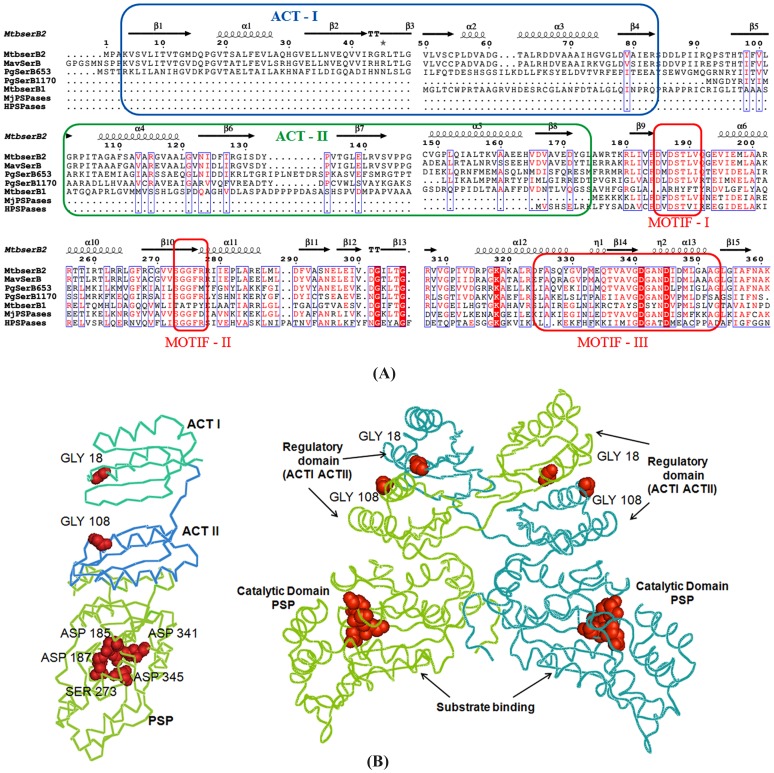
Sequence alignment and modeling. (**A**) **Sequence alignment of MtSerB2** with sequences of Phosphoserine phosphatases from *M. avium* (MavSerB), *P. gingivalis* (PgSerB653), *P. gingivalis* (PgSerB1170), *M. tuberculosis* (MtSerB1), MjPSPase (*M. janaschii*) and HsPSPase *(Homo sapiens)*. Three conserved motifs of the PSP domain are shown in *red*. The ACT1 and ACT2 domains are colored *blue* and *green* respectively Secondary structural elements are also indicated. The sequences highlighted in *red* represents high consensus whereas those in *blue* represents low consensus (**B**) **Modeled structure of MtSerB2**. MtSerB2 structure was modeled using *M. avium* SerB structure (PDB: 3P96) and Modeler 9.10. The monomeric and dimeric associations are depicted and the individual domains are labelled. Gly residues important for binding ligands in ACT domains are shown in ‘stick’ representation and labeled for clarity. Selected catalytic residues on the PSP domain are also labeled and depicted.

*M. tuberculosis H37Rv* contains two phosphoserine phosphatases (E.C. 3.1.3.3; systematic name: O-phosphoserinephosphohydrolase). One of these, MtSerB1, Rv0505, contains a classic phosphoserine phosphatase domain (PSP) while the other one, MtSerB2 (Rv3042c), is unusual and contains two ACT (Aspartate kinase, Chorismate mutase, and TyrA protein regulatory domain) domains in tandem at the N-terminus followed by a phosphoserine phosphatase domain. ACT domains (Pfam 01842) are small- molecule binding domains consisting of ∼70–80 amino acids. This domain functions as a common regulatory element and has been implicated in the control of metabolism, solute transport, and signal transduction, amongst others [1]–[3]. Transposon mutagenesis experiments have identified that MtSerB2 is essential for the pathogen's viability while MtSerB1 is not [4]. SerB proteins belong to the Haloacid dehalogenases (HAD) family, a relatively less-studied enzyme family that is involved in various metabolic processes [3], [5]–[11]. The latter proteins exhibit low sequence similarity among themselves and are characterized by the presence of three conserved motifs (**Fig. 1A**).

Phosphoserine phosphatases (E.C. 3.1.3.3) catalyze the reaction: O-phospho-L (or D)- Serine + H_2_O  =  L (or D)-Serine + Phosphate.

Several enzymes, that correspond to only the PSP domain, have been characterized structurally and functionally from various sources including those from *M. jannaschii* [12], *H. sapiens* [13], [14], *H. pylori* (PDB ID 3M1Y, unpublished data) and *V. cholerae* (PDB ID 3N28, unpublished data). The reported work has revealed several details of the mechanistic action in these proteins including interactions with transition state analogs [15].

Recently, an enzyme (SerB653) from *P. gingivalis*, similar in architecture to MtSerB2, was shown to be important for invasion. Additionally, it interacts with several human phosphoproteins. *P. gingivalis* is an opportunistic, invasive pathogen where invasion requires epithelial cell microfilament and microtubule rearrangements. In this context, it has been shown that exogenously added *P. gingivalis* SerB653 protein induced microtubule rearrangements in HIGK cells (human immortalized gingival keratinocytes) [16]. The studies concluded that *P. gingivalis* SerB653 acts like an invasin.

Presently, we demonstrate that *M. tuberculosis* SerB2 is a member of the HAD enzyme family. The PSP domain contains the three conserved sequence motifs that characterize classical PSPases. The enzyme requires a divalent metal ion co-factor for activity. On the other hand, the binding of amino acids to the enzyme, either enhances/reduces/has no effect on its activity. Very recently, the crystal structure of the *M. avium* homolog in the *apo* form was solved as part of the Seattle structural genomics initiative, although no characterization was carried out [17]. Given the high sequence homology between the *M. tuberculosis* and the *M. avium* enzymes, we could rationalize the characterization results based on the *M. avium* structure. Inhibition studies involving a variety of compounds, backed by *in silico* docking experiments, suggests that amino acids like Ser mainly bind to sites on the ACT domains while other inhibitors like Sodium vanadate and NaF bind to the PSP domain alone. The latter results suggest that it is possible to inhibit the activity of the protein through the design of inhibitors that specifically bind to the ACT domains and not just the PSP domain. Further, we find that exogenously added MtSerB2 induces microtubule rearrangements in THP-1 cells, a cell- line that can differentiate into macrophage-like cells. The experiments with mutant MtSerB2 demonstrates that the phosphatase activity is co-related to the elicited microtubule rearrangements.

## Experimental Procedures

### Cloning & over-expression of MtSerB2 (Rv3042c), its subunits and mutants

Primers (MWG) to clone MtSerB2 and its variants contain BamHI and HindIII restriction sites in the forward and reverse primers respectively. PCR reactions were carried out using Pfx DNA polymerase (*Invitrogen*). The product corresponding to the native enzyme was cloned into *pET*23a vector (*Novagen*) and called *pET*23a:MtSerB2. Recombinant Hexa-his-tagged MtSerB2 was expressed using *E. coli* C41 (*DE3*). The pET23a:SerB2_ACTD (residues 1–165) and pET23a:SerB2_PSPD (residues 165–409) constructs corresponding to the ACT and PSP domain mutants were used to over-express the mutants in *E. coli* BL21 (DE3) and C41 strains respectively. Other mutants that were generated are G18A, G108A, D185N, D187N, S273A, K318A, D341N, D345N, D185N/D187N & D341N/D347N. Mutants were over-expressed similar to the wild-type except that D187N and S273A were grown at lower temperature i.e., 25°C instead of 37°C to overcome problems of solubility of protein. Integrity of all constructs was verified by sequencing.

### Purification of proteins

1L LB medium containing 100 µg/ml ampicillin was inoculated with 1% seed culture. It was then grown overnight at 37°C, with 180 rpm until ∼0.6 OD_600_. Protein expression was then induced by adding 0.5 mM IPTG and the culture was grown further for 8 hrs at 37°C, 120 rpm. Subsequently, cells were harvested, resuspended in buffer A (50 mM Tris-HCl, pH 8.0, 200 mM NaCl, 5 mM imidazole and 12% glycerol) and lysed by sonication after the addition of 1 mM of phenyl methyl sulphonyl fluoride. A Ni^++^-IDA column (*GE Healthcare*) pre-equilibrated with buffer A was used for purification. Protein was eluted using a linear Imidazole gradient to 1 M in buffer B (50 mM Tris-HCl, pH 8.0 and 200 mM NaCl). Fractions were pooled after SDS-PAGE analysis and precipitated using Ammonium sulfate (40%). Pellet was resuspended in 50 mM Tris-HCl, pH 8.0, 50 mM NaCl, 5 mM β-mercaptoethanol (Buffer C) and further applied onto Superdex S200 (*GE Healthcare*) gel-filtration column pre-equilibrated with buffer C. Protein was pooled and concentrated to 20 mg/ml using 10-kDa cutoff centricons (*Amicon*). Concentration of proteins were determined using the Bradford reagent [18] with BSA as standard. Proteins remained stable at 4°C without degradation for weeks. Purity was confirmed on 12% SDS-PAGE gels. ACT and PSP domains were expressed in media supplemented with 10% glycerol at 37°C and purified using the same buffers at pH 7.5 as above except that they were supplemented with 10% glycerol.

### Homology modeling and docking studies

Multiple sequence alignments were carried out using *Multalign* [19] and sequence analysis figures were generated using ESPript 2.2 [20]. Neighbour-joining phylogenetic tree of phosphoserine phosphatases was calculated using ClustalX [21]. BLAST [22] search against the NCBI database with MtSerB2 revealed highest similarity (83%) with *M. avium* SerB whose X-ray structure has recently been reported [17]. Homology models of MtSerB2 based on *M. avium* SerB (PDB code 3P96) were generated using Modeller9.10 (http://salilab.org/modeller/) [23]. Geometry of the models were checked using *Procheck* [24]. 3D structures of all the compounds were constructed using the Builder module of InsightII version 2000 (Accelrys, San Diego, CA). The respective geometries of the compounds were subsequently optimized, with the maximum number of iterations set to 1000 and the convergence criterion set to 0.01 Kcal/mol respectively. The protein has a total of three binding pockets, located in the ACTI, ACTII and PSP domains respectively. Docking calculations were performed using *Autodock3.0.*5 [25] and analyzed using AutoDock Tools and PyMOL (v.1.2r3pre; Schrodinger LLC).

### Hydrolytic activity of MtSerB2 and its variants using malachite green

Hydrolytic activity of SerB2 was measured at various temperatures by following the release of Pi from l- 3-phosphoserine and expressed as nmol P_i_/min/mg of SerB2. Assays were performed in triplicate in mixtures (200 µl) containing 20 mM Tris-HCl (pH 7.5), 5 mM MgCl_2_, 1 mM DTT, 100 nM MtSerB2 and l-3-phosphoserine. Inhibition studies involving L- AP3, DL- AP3, l-serine, Chlorpromazine hydrochloride and Fluoride were also carried out. Reactions were initiated by adding 100 nM MtSerB2 and incubated at 37°C. They were terminated after 30 min by adding 200 µl freshly prepared malachite green-ammonium molybdate dye reagent. After 1 min at room temperature, 10 µl of 34% citric acid was added to stop the reaction and absorbance at 630 nm was measured [26]. Amounts of released inorganic phosphate in triplicate samples were measured photometrically by referring to a standard curve, which was prepared with dilutions of a standard solution of inorganic phosphate. For assays involving externally added amino acids, native enzyme was incubated with the reaction mixture (25 mM Tris-HCl pH 7.5, 5 mM MgCl_2_, 1 mM DTT and 0.2% BSA) and varying concentration of amino acids for one hour at 37°C. Reactions were started by adding 1 mM l- phosphoserine and were incubated for 30 min at 37°C. Inorganic phosphate released was quantified spectroscopically using malachite green as before.

### Isothermal Titration Microcalorimetry

ITC experiments were performed using a VP-ITC instrument (*GE*). Injections of 6 µl of substrate solution were added from a computer-controlled micro syringe at 3 min intervals into the protein solution (cell volume = 1.43 ml) with stirring at 350 rpm. The concentration of the protein was 30 µM and the substrate was 0.6 mM. Titrations were done at pH 7.4 using 50 mM sodium phosphate and 50 mM NaCl. Experimental data were fitted to a theoretical titration curve using software supplied by Microcal, with ΔH (binding enthalpy kcal mol- ^1^), Ka (association constant) and n (number of binding sites per monomer), as adjustable parameters. The quantity c = K_a_M_t_, where M_t_ is the initial macromolecule concentration, is of importance in titration microcalorimetry [27]. All experiments were performed with c values 1<c<200. The instrument was calibrated using the calibration kit supplied by the manufacturer. Thermodynamic parameters were calculated from the Gibbs free energy equation.

### Fluorescent Microscopy

THP-1 human macrophage cell lines were acquired from the American Type Culture Collection, USA and cultured in RPMI 1640 medium supplemented with 10% fetal calf serum at 37°C and 5% CO_2_. Cells were pelleted by centrifugation at 100xg for 10 min and resuspended in fresh complete medium. Cells were treated with 20 nM PMA and seeded at a density of 10^6^ cells/well in 12-well plates and incubated for 16 h to prepare the monolayer of macrophages. To observe the changes in the microtubules, the macrophages were treated with 100 µg of purified proteins of SerB2, SerB2 mutant D341N, SerB2 PSP domain and SerB2 ACT domain for 30 minutes and 2 hours respectively. The monolayer was washed three times with incomplete medium and fixed with ice-cold methanol. Cells were further incubated with α-Tubulin (4G1: sc-58666, Santa Cruz inc.) antibody at 1∶1000 dilution for 1 h at 37°C, followed by secondary antibody goat anti-mouse IgG1 (sc-2979, Santa Cruz inc.) conjugated with Texas red at 1∶200 for 1 h in the dark at 37°C. Images were captured at 40X and 100X with a Nikon Eclipse E400 fluorescent microscope.

## Results

### Sequence analysis of MtSerB2 suggests that it is an unusual phosphoserine phosphatase

*M. tuberculosis* Rv3042c was identified as a SerB2 protein belonging to the HAD family of hydrolases. The protein folds into three domains, *viz.* two ACT domains occurring in tandem at the N-terminus followed by the classical phosphatase domain (**Fig. 1A**). Each ACT domain adopts a β_1_α_1_β_2_β_3_α_2_β_4_ fold and is characterized by the presence of an invariant Gly residue at the turn between the β_1_ sheet and α_1_ helix [1]–[3], [28]. This Gly is important for the binding of small-molecules to the ACT domain. In MtSerB2, the important Gly in the two ACT-domains are G18 and G108 respectively (**Fig. 1**; **S1 Figure**). *M. tuberculosis* contains another predicted HAD family SerB enzyme, Rv0505c (MtSerB1). MtSerB2 and MtSerB1 proteins are 24% identical overall and 29% similar in the PSP domain. In expression studies, MtSerB1 was found to be insoluble under various tested conditions and we subsequently characterized MtSerB2. Structural comparisons involving the *M. avium* SerB (PDB: 3P96) and modeled MtSerB2 (**Fig. 1B**) shows that the ACT domains exhibit extensive interactions in the dimer and in fact take part in domain-swapping in the oligomer.

### MtSerB2 is specific for l- phosphoserine and its activity is modulated by ACT domains

We tested various substrates against MtSerB2 in activity assays. The protein exhibits high specificity for l-phophoserine compared to substrates like l- phosphotyrosine and l-phosphothreonine. Percentage activity and final docked energies with respect to l-phosphoserine are tabulated (**Table 1**). The relative activity for l-phosphoserine is 100% while that against l- phosphothreonine is only ∼5%. This result contrasts with the specificities exhibited by other characterized enzymes, *e.g*. *H. sapiens* and *M. jannaschii* phosphoserine phosphatases. The latter enzymes use all phospho- amino acids like l- phosphoserine, l-phosphotyrosine and l-phosphothreonine, albeit with different efficacies with the exception of *P. gingivalis* SerB653 that is specific for l-phosphoserine as a substrate.

**Table 1:** Relative activity (%) and *in silico* docking energy of different substrates.

Substrates	Relative activity (%)	Free energy (Kcal/mol)
l-Phosphoserine	100	−16.8
l-Phosphothreonine	5.1	−11.9
l-Phosphotyrosine	1.7	−4.5
Glucose-6-phosphate	1.4	−6
ATP	5.5	25.9

**Figure 2:**
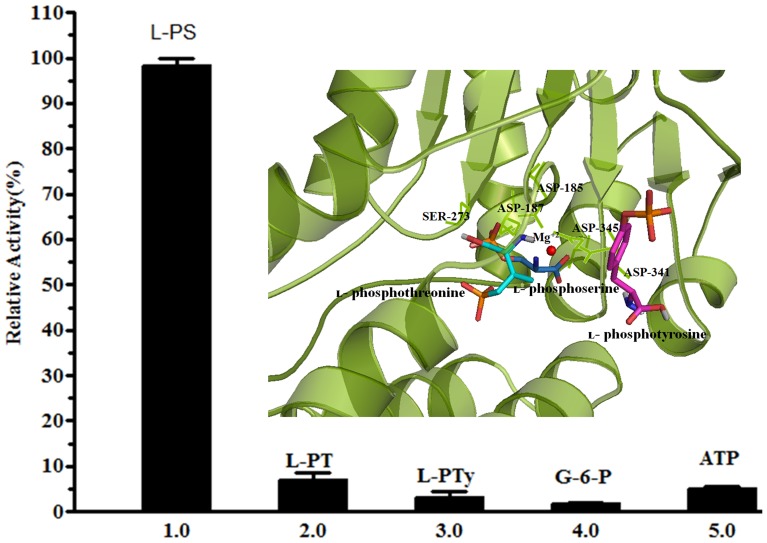
Substrate specificity of MtSerB2. Relative change in the hydrolysis of different substrates. l- PS depicts l-phosphoserine, l- PT depicts l-phosphothreonine, l- PTy depicts l-phosphotyrosine, G-6-P depicts Glucose-6-phosphate and ATP is adenosine triphosphate. The experiment was performed in triplicate and the values represent the average. Inset shows a close-up of the active site and the docked moiety is indicated in stick representation. l-phosphotyrosine is occluded from the active site due to steric hindrance while l-phosphoserine fits well in the active site.

We rationalized the substrate specificity of MtSerB2 through *in silico* docking studies (**Fig. 2**). We find that although l-phosphothreonine can also fit into the active site, the orientation of the threonine moiety is opposite to that of l- phosphoserine. This will conceivably prevent the formation of the phosphoaspartyl bond with the active site D185 that is necessary for the hydrolysis of the substrate. Analogously, inhibition effects of calcium on enzyme activity have been previously attributed to the Asp residue being precluded from forming the phosphoaspartyl bond with the substrate [29]. On the other hand, l- phosphotyrosine is occluded from the active site due to steric hindrance, and this can be attributed to the larger side chain of tyrosine. The docking results support the specificity of MtSerB2 for l-phosphoserine. We also found that the protein does not utilize substrates that contain a phosphate-ester bond such as glucose-6-phosphate and ATP.

**Figure 3:**
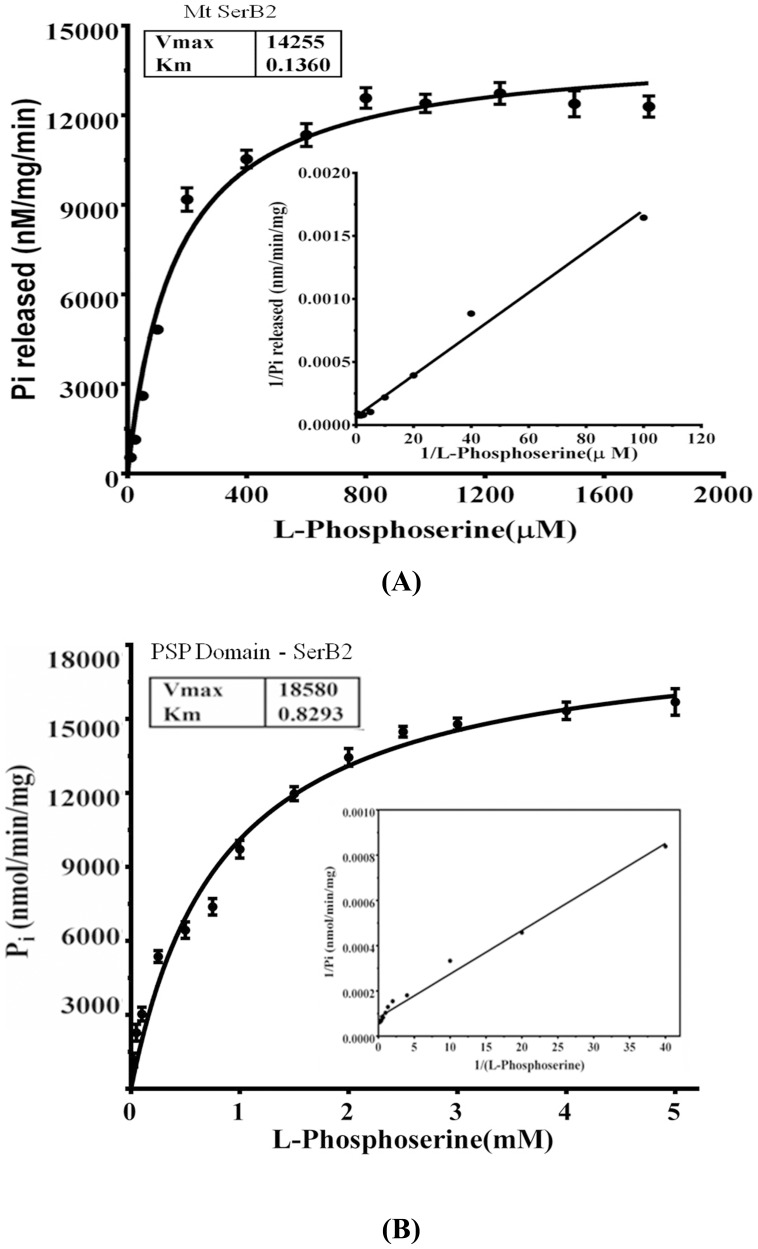
Determination of K_m_ and K_cat_ values for l-phosphoserine. Michaelis-Menten plots calculated using l-phosphoserine as the substrate, for (**A**) MtSerB2 and (**B**) PSPD respectively. Inset shows double-reciprocal plots of the initial velocities (1/Vo) against the reciprocal of l-phosphoserine phosphate. The experiment was performed in triplicate and the values represent their average.

Activity assays involving the PSP domain alone (residues 166–409) were also carried out. We found that the PSP domain itself is capable of hydrolyzing l-phosphoserine, albeit with much reduced efficacy (**Table 2** **and** **Fig. 3A, B**). The turnover number decreases by about 3-fold (0.841×10^4^) compared to that of the full- length enzyme (2.54×10^4^). Additionally, the K_m_ for PSP domain increases by ∼6 times compared to that for the full- length protein. We conclude that l-phosphoserine has much reduced affinity for the PSP domain alone, and attribute the higher substrate affinity of full-length MtSerB2 for l-phosphoserine to sites on the respective ACT domains. We also found that there is large decrease in the catalytic efficiency, of the order of 10^5^, in the PSP domain alone. However this activity loss is substantially reversed (∼40% of that of the full- length enzyme) by the equimolar addition of the purified ACT domains. Also, adding the ACT domain only marginally increases the K_m_ (∼1.8 times) compared to that of the full- length enzyme. The above results demonstrate that the ACT domains play an important role in modulating the activity of MtSerB2.

**Table 2:** Kinetic parameters of MtSerB2 and its mutants.

Enzyme	V_max_ (nM/min/mg)	K_m_ (µ M)	K_cat_ (sec^−1^) ×10^4^	K_cat_/K_m_×10^10^ (M^−1^sec^−1^)
Wild-type	14250±576	135.9±11.3	2.54	0.0187
G18A	8829±451	208.2±15.3	1.65	0.0079
G108A	9666±843	259±19.6	1.56	0.0060
D185N	ND	ND	ND	ND
D187N	11897±1147	49.1±6.5	1.94	0.0395
S273A	ND	ND	ND	ND
K318A	7288±538	125.9±8.6	1.13	0.0089
D341N	2062±149	221.7±14.7	0.37	0.0016
D345N	6345±349	50.8±5.6	1.23	0.0242
D185N/D187N	ND	ND	ND	ND
D341N/D345N	ND	ND	ND	ND
PSPD	18581±1770	829.9±77.0	0.84	0.00101
PSPD + ACTD	10390±242	242.6±39.1	1.68	0.0069

ND, not determinable.

### MtSerB2 is a divalent metal- ion dependent alkaline phosphatase that is highly active near neutral pH and physiological temperature

**Figure 4:**
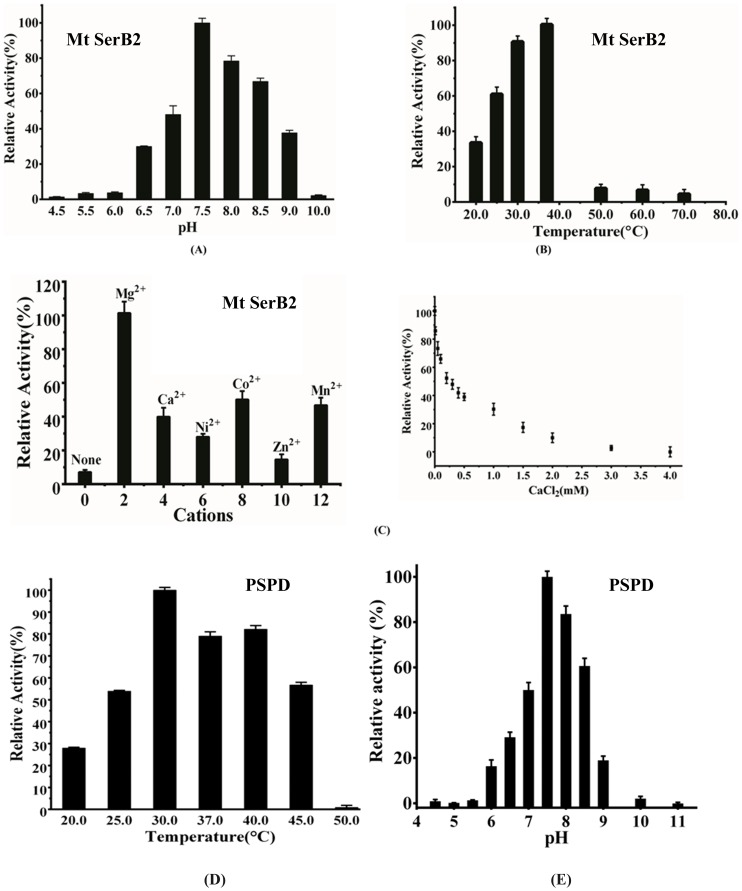
Effect of various factors on functional properties of MtSerB2 and its phosphatase domain (PSPD) respectively. (**A**) Relative change in hydrolysis of l-phosphoserine with increasing pH. Hydrolysis at pH 7.5 was taken as 100%. (**B**) Changes in the enzyme activity of MtSerB2 on increasing the temperature. Data are shown in percentages with enzyme activity observed for MtSerB2 at 37°C taken as 100%. (**C**) Effect of divalent cations on enzymatic hydrolysis of l-phosphoserine by MtSerB2. Relative activity was measured at concentrations of different divalent cations and that with Mg^2+^ was considered as 100%. Inset shows inhibition by Calcium Chloride. (**D**) Effect of varying the temperature on the phosphatase activity of PSPD. (**E**) Optimal pH for maximum substrate hydrolysis.

The hydrolytic activity profile of MtSerB2 was checked in the pH range 4.5–10. MtSerB2 at optimal pH 7.5 has 20% greater activity than at 8.0 (**Fig. 4A**). Activity declined progressively before pH 7.5 and was almost abolished at 6.0 while at higher pH MtSerB2 remained active till pH 9.0. The temperature-dependence of the activity was also probed (**Fig. 4B**). MtSerB2 exhibits maximum activity at 37°C. It declines at higher temperatures and is completely abolished by 50°C while in case of PSP domain, maximum activity was found at 30°C and that activity was completely abolished at 50°C (**Fig. 4D**). We also similarly checked the hydrolytic activity profile of the PSP domain mutant *vis-a-vis* the pH. The pH dependence of the activity of PSP domain was found to be similar to that of the full- length enzyme and the optimal pH is pH 7.5, with greater than 15% activity observed at 8.0 (**Fig. 4E**).

A characteristic of HAD family phosphatases is their dependence on divalent cations. Addition of sodium fluoride or EDTA to either phosphatase reaction resulted in a decrease of enzyme activity. The results show that similar to homologs from humans and *Methanococcus*, MtSerB2 is Mg^2+^-dependent. The presence of Mg^2+^ plausibly balances the negative charge of the catalytic pocket that contains three Asp residues. In human phosphoserine phosphatase, other metal cations like Mn^2+^ and Co^2+^ also act as activators. However, in the case of MtSerB2 all other tested divalent cations like Ca^2+^, Ni^2+^, Co^2+^, Mn^2+^ and Zn^2+^ deactivates it (**Fig. 4C**). In fact, Zn^2+^ inhibits the activity at nanomolar levels. It has been earlier suggested (28) that Ca^2+^-dependent inhibition is apparently due to its larger size compared to Mg^2+^, that enables it to co-ordinate with both oxygen atoms of the catalytic site's Asp185. Similar reasons could explain the observed Zn^2+^-dependent inhibition. The results show that MtSerB2 is a robust, divalent cation dependent alkaline phosphatase.

### Mutational analysis of residues in the catalytic site motifs of MtSerB2

HAD-family phosphatases are characterized by three motifs in the catalytic site. The motif 1, (DXDX(T/V)), is characterized by a highly conserved Asp at the first position that is probably involved in the formation of the phosphoaspartate intermediate. The second motif, S/TXX, contains an essential ser/thr residue, while the third motif, K-(X) 18-30-(G/S)(D/S)XXX(D/N), contains the important lys and asp residues [5], [12]. We mutated residues in these motifs to probe their roles. Previous studies involving the active site in human PSP [30], has shown that D20, D22, S109, K158, D179 and D183 residues are important for enzymatic hydrolysis. Corresponding amino acids in MtSerB2 are D185, D187, S273, K318, D341 and D345. **Table 2** lists the various parameters of the respective mutants. Some of the mutants caused a moderate decrease in activity, while other mutants like D185N, D185N/D187N, S273A and D341N/D345N inactivated the enzyme almost 100%. The increase in K_m_ value for phosphoserine observed in D341N suggests that this amino acid participates in substrate binding. Previous mutational analysis of residues in the first motif in *M. jannaschii* [12] showed that D185 could not be substituted by N without complete loss of activity, whereas the replacement of D187 by N allowed the retention of about 80% of the activity. This agrees with the critical role played by D185 in the formation of the phosphoenzyme intermediate. In the second motif, S273 is conserved in the superfamily as S or N. The presence of the hydroxyl group on S273 seems particularly important since the S273A mutation results in complete loss of hydrolytic activity. On the other hand, in other members of the superfamily, the activity decreases but is not abolished. In human phosphoserine phosphatase, there is almost complete loss of activity when the first conserved Asp residue in the third motif (DXXXD) is replaced by R [30]. Replacement of the highly conserved D341 in the third motif by residues other than E results in a near complete loss of activity in human phosphoserine phosphatase, halo acid dehalogenase, and Ca^2+^ATPase [29]. In the case of halo acid dehalogenase, the residue was proposed to play a role in activating a water molecule that would be used in hydrolysis of the covalent intermediate. Furthermore, the fact that mutation of D341 (D183 in humans) in phosphoserine phosphatase or the equivalent residue in Ca^2+^ATPase by N abolishes the formation of the phospho enzyme indicates that this aspartate plays a major role in this process. The activity of the D345 mutant gets reduced (>40%) but is not completely abolished. The double mutant D341N/D345N however, becomes inactive. The mutational analysis shows that MtSerB2 is a HAD family protein with interesting differences compared to human phosphoserine phosphatase.

### Inhibition of MtSerB2 activity, comparison with PSP Domain, and in silico rationalization

MtSerB2 is an essential protein, and represents a novel target for development of therapeutics with new modes of action. We therefore examined, in the first place, efficacy of known inhibitors of phosphatases. The preliminary results could then form the foundation for the development of more robust inhibitors using various structure based strategies including similarity searches. Accordingly a variety of inhibitors, some of which have earlier been reported [31] to be good inhibitors of phosphatases, were tested. Sodium vanadate and Okadaic acid are known potent inhibitors of phosphatases. However, in the case of MtSerB2, Okadaic acid is not able to inhibit the phosphatase activity. The IC_50_ values and I_MAX_ values, *i.e* difference between minimum and maximum inhibition, of these inhibitors are listed in **Table 3**. L- AP3 (L-2-amino-3-phosphonopropionate), DL- AP3 (DL- 2-Amino-3-Phosphonopropionic Acid), l-serine, Chlorpromazine, α-Glycerophosphorylcholine, phosphorylcholine and Sodium fluoride inhibit the hydrolysis of l- phosphoserine but exhibit differences in the relative potencies compared to other phosphatases. Using l- phosphoserine as the substrate, the most potent inhibitor was l- serine (IC_50_ = 0.78 µM) followed by Chlorpromazine (IC_50_ = 0.92 µM). The rank order of potency for inhibition was l-serine> Chlorpromazine> Sodium vanadate> Fluorides>L- AP3>D, L AP3> α-Glycerophosphorylcholine> Phosphorylcholine. A plot of titration of inhibition by L-serine is in S2 **Figure**.

**Table 3:** Comparative inhibition studies of MtSerB2 and the PSP domain using l- phosphoserine as the substrate.

Inhibitor	*SerB2*	*PSPD*
	IC_50_ (µM)	Imax (%)	IC_50_ (µM)	Imax (%)
l-serine	0.78±0.08	65	823.7±39	70
				
Chlorpromazine	0.92±0.1	70	6.25±0.3	95
				
Sodium Vanadate	2536±198	88	2235±150	93
				
NaF	3107±155	55	2782±216	72
				
D,L- AP3	ND	22	ND	19
				
L- AP3	ND	23	ND	21
				
α-GPC	ND	35	ND	31
				
Phosphorylcholine	ND	10	ND	14
				
Okadaic acid	ND	14	ND	22
				

α-GPC: α-Glycerophosphorylcholine, Chlorpromazine: Chlorpromazine Hydrochloride, ND: Not possible to determine.

**Figure 5:**
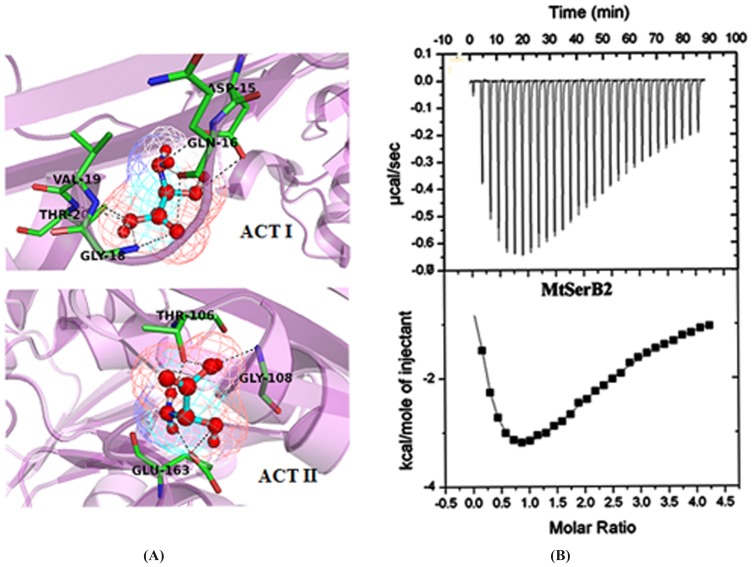
Molecular Docking and Isothermal calorimetry experiments. (**A**) **Docking modes of l-serine with MtSerB2**. l-serine was docked against the binding sites predicted in the ACTI and ACTII domains respectively. Interactions of l-serine with ACTI (*top*) and ACTII (*bottom*) domains are shown here. Key interacting residues are labeled in *black* and shown in stick representation while the rest of the site is shown in cartoon representation. l-serine is depicted in ball- and-stick representation. (**B**) **ITC experiments involving interactions of l-serine with MtSerB2**. Titration of l-serine (300 µM) into MtSerB2 solution (30 µM). The experiments were performed in 50 mM sodium phosphate buffer, pH 7.4, and 50 mM NaCl and 2 mM β mercaptoethanol at 25°C. The cell volume was 1.43 ml while the injection volume was 6 µl.

The inhibition studies were also carried out against the PSP domain (**Table 3**) to compare with the full-length protein. l-serine is a feedback inhibitor, and accordingly the activity of PSP domain was determined in the presence of increasing concentration of l-serine, and also with other inhibitors. Chlorpromazine hydrochloride exhibited inhibition of the PSP domain with IC_50_ ∼6.25 µM. *In silico* docking experiments involving Chlorpromazine suggest two different interaction modes for the molecule. One of the orientations is similar to that of the other inhibitors in the active site, while a second molecule was found to interact with Arg177 located away from the active site. The alternate predicted modes are in line with the observed non-competitive inhibition exhibited by chlorpromazine. Sodium vanadate (IC_50_∼2.5 mM) and NaF (∼3.0 mM) also exhibited similar inhibition, albeit weak, of both constructs suggesting that they bind primarily to the PSP domain. Surprisingly, l- serine exhibited a drastic drop (IC_50_ 823.7 µM) in the inhibition of the PSP domain as compared to the inhibition of the full-length protein. As is detailed in the next sections, this difference is likely because l-serine, unlike the other tested compounds, binds to the ACT domains. *In silico* docking experiments support that l-serine, binds strongly to the ACTI and ACTII domains of MtSerB2 compared to other inhibitors (**Fig. 5A**), a result that is in line with the above experiments.

### Identification of a specific l-serine mediated oligomeric transition

Since ACT domains are known to bind amino acids, it was natural to examine the effects of different amino acids on the activity of the native enzyme. We checked the effects of various amino acids on the hydrolysis of l-phosphoserine (**Table 4**). We found that some amino acids act as inhibitors, some as activators, and others exhibit neutral effects. We initially tested Ser, Gly and Thr, interestingly, other than Ser, we found that Gly and Thr also inhibit enzyme activity. The respective effects of activation of enzyme activity by Lys and Phe and the inhibitory effects of Pro, Gly, Glu, Arg, Ala, His, Ser and Trp are also tabulated. Additionally, Trp (IC_50_ = 320 µM) was also found to be a strong inhibitor. On the other hand, Lys (40%) was found to be the strongest activator.

**Table 4:** Effects of added amino acids on MtSerB2 phosphatase activity.

Aminoacid	% activation or IC_50_ (µM)	Activation factor
Lys	40%	Activation
Tyr	38%	Activation
Phe	35%	Activation
Ser	0.78±0.1	Inhibition
Trp	321.7±23	Inhibition
Glu	363±20	Inhibition
Gly	381.3±15	Inhibition
Gln	432±13	Inhibition
Arg	632.3±14	Inhibition
Asp	1616±84	Inhibitor
Thr	3463±206	Inhibition
Val	3663±212	Inhibition
Ala	3828±234	Inhibition
His	4861±218	Inhibition
Pro	>5 mM	Inhibition
Met	No Change	Non modulator
Ile	No Change	Non modulator
Leu	No Change	Non modulator
Asn	No Change	Non modulator
Cys	No Change	Non Modulator

The tabulated values represent the average of three independent experiments. Activation is represented as percentages while the IC_50_ values are given where inhibition was observed.

We subsequently looked to measure the affinity of MtSerB2 for various ligands/amino acids. The affinity of l-serine, that exhibited the highest inhibitory effect, was probed through Isothermal calorimetry (ITC). The earlier delineated inhibition results suggested that l-serine should bind to all 3 small molecule binding sites in MtSerB2. A typical ITC isotherm produced by titrations of l- serine into MtSerB2 is shown in **Fig. 5B**. Attempts to fit the curves to models containing upto 6 ligands per protein molecule, assuming a dimer, did not yield conclusive quantitative results. However, some qualitative features of the interactions between l-serine and the protein can be inferred. At first glance the curve is suggestive of cooperative binding and the addition of ligand in the early stages results in increasing heats of binding, followed by saturation of the protein by ligand. On the other hand, the Hill co-efficient values did not suggest strong co-operativity. We therefore examined other possibilities, including the change in the oligomerization status of the protein.

**Figure 6:**
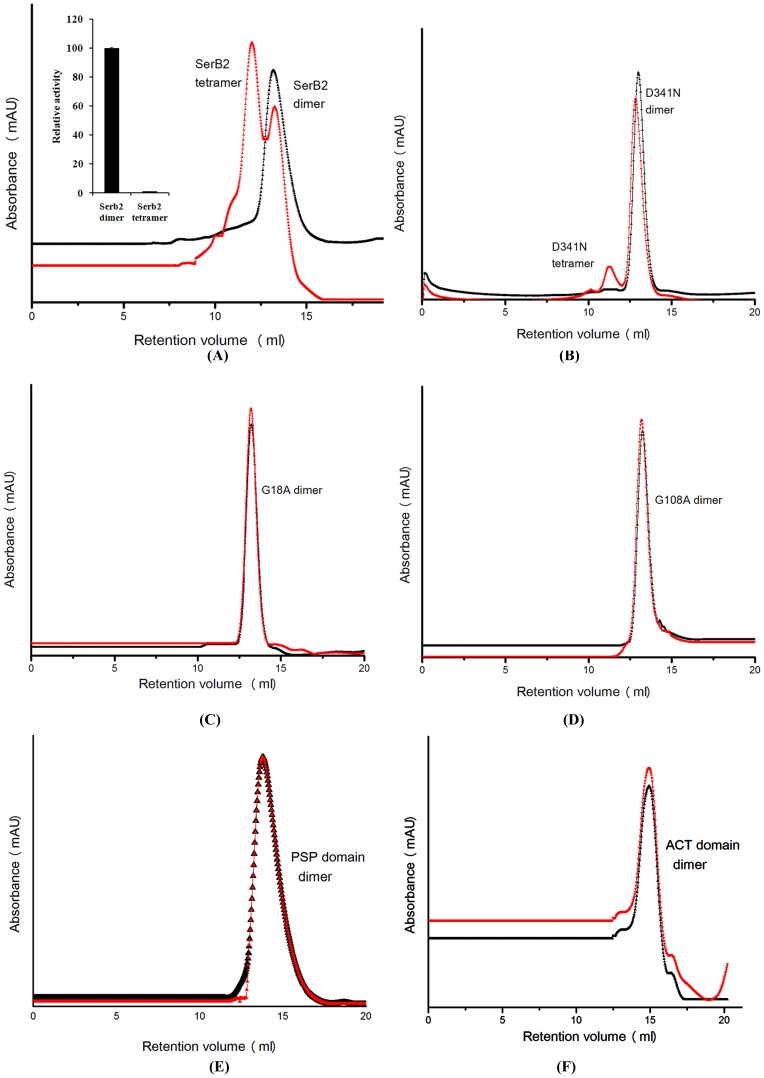
Size exclusion chromatography experiments involving MtSerB2, its mutants and l-serine. (**A**) **Wildtype SerB2** (**B**) **D341N** (**C**) **G18A** (D) **G108A**. Chromatogram in the absence of l-serine is in *black*, whereas the chromatogram in the presence of of l-serine, and MtSerB2 and its mutants, are shown in grey. Wild-type MtSerB2 and D341N show a shift to the tetrameric/higher order oligomeric forms in the presence of ∼0.8 molar ratio of l-serine.

**Figure 7:**
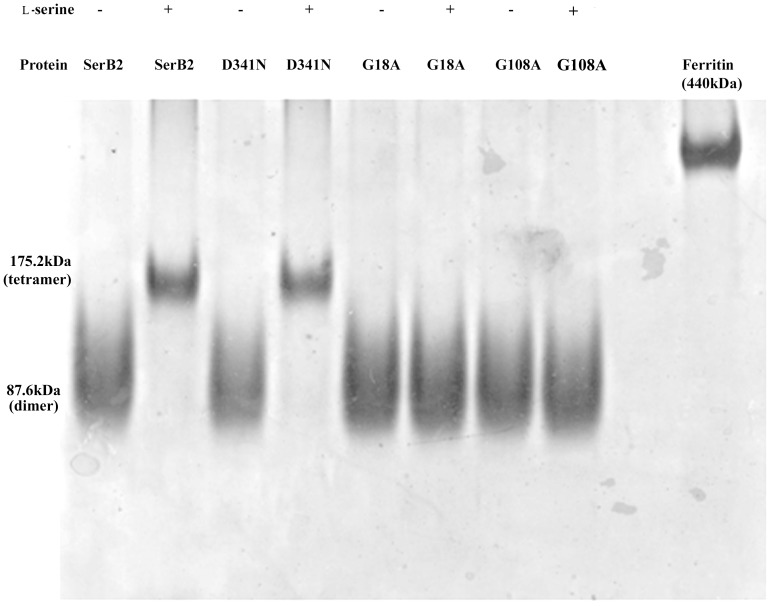
Native PAGE. The appropriate fractions from the size-exclusion chromatography experiments were evaluated using Native PAGE. Clearly MtSerB2 and the D341N active-site mutant shifted to a tetrameric association in the presence of ∼0.8 molar ratio of l-serine. On the other hand, the G18A and G108A ACT-domain mutants exhibit no such transition.

We accordingly carried out analytical size exclusion chromatography experiments using a Superdex S200 (*GE Healthcare*) gel-filtration column calibrated with low and high molecular weight range markers (S3 **Figure**). The column was equilibrated with buffer containing 50 mM Tris-HCl, pH 8.0, 50 mM NaCl, 5 mM β-mercaptoethanol and supplemented with required molar-ratio of Ser for the respective experiments. The samples were pre-equilibrated with the required amino acid concentration for 1 h. (**Fig. 6**). We found that the dimeric population of MtSerB2 shifts to a tetramer (higher order oligomer) in the presence of ∼0.8 molar ratio of l-serine and MtSerB2. A look at the ITC experiments shows that it is consistent with the l-serine mediated oligomeric transition and the transition in the ITC results is also at ∼0.8 molar ratio of l-serine. We subsequently performed analytical size exclusion chromatography experiments with the G18A and G108A mutants. The latter mutants do not bind l-serine and correspondingly no oligomeric transitions were observed in the presence of l-serine. On the other hand, the D341N catalytic site mutant exhibits the transition, albeit weaker, supporting that the ACT domains play an important role in the observed oligomeric transition. Further, it supports that binding of l-serine to the latter domains is important. Additionally, we carried out activity assays against the higher order oligomeric form and found that this is inactive (**Fig. 6A** **inset**). Native PAGE corresponding to the size-exclusion-chromatography fractions of MtSerB2, G18A and G108A, in the presence and absence of l-serine, were also evaluated (**Fig. 7**). The latter experiment independently confirms the size exclusion chromatography results. Overall, the above results suggest a functionally relevant feedback between the oligomeric state and binding of l-serine. Increased concentration of the inhibitor shifts the protein into an inactive higher order oligomeric form.

### Effects of exogenously added MtSerB2 on human THP-1 cells

**Figure 8:**
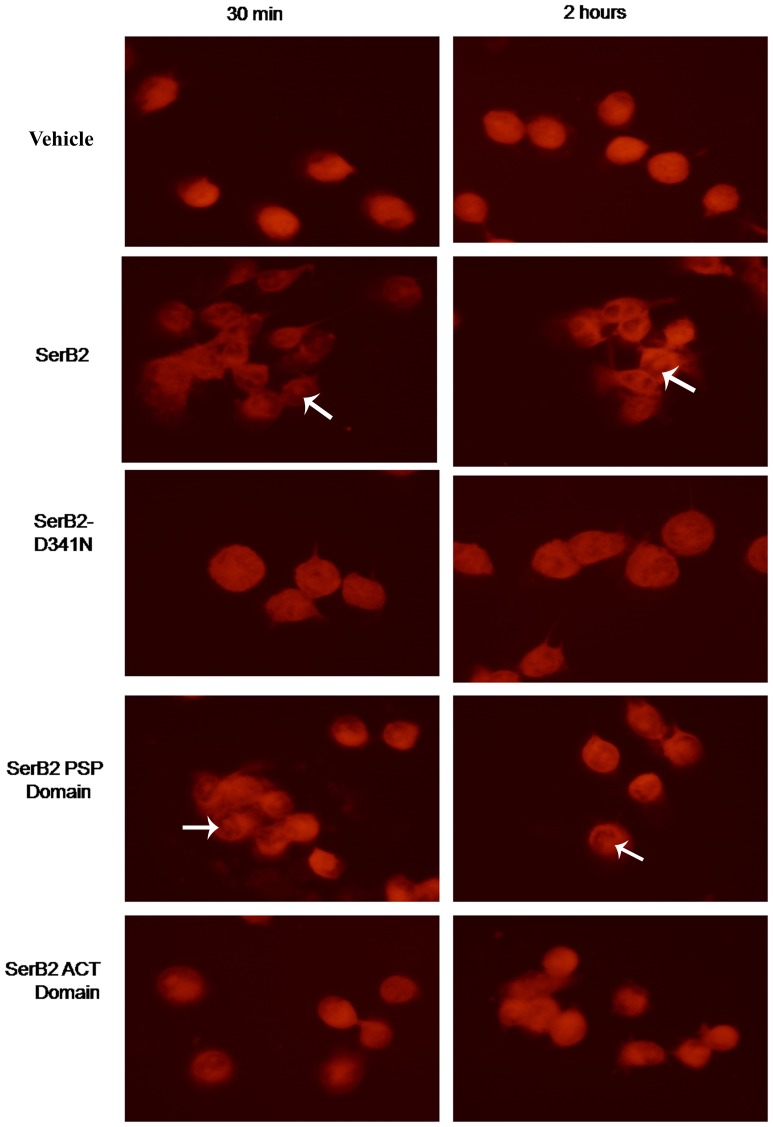
Fluorescent microscopy experiments involving THP-1 cells shows alteration in cell microtubules in the presence of MtSerB2. Exogenous addition of purified MtSerB2 protein to THP-1 cells induces microtubule rearrangements. Cells were stained for α-tubulin (*red*) and images were taken at 60× magnification. Arrows indicate the presence of enriched tubulin at the cell surface. Controls (vehicle) contained enzyme buffer only. Additional controls involve the D341N catalytic site mutant which induced very less/negligible tubulin rearrangement and this is attributed to the residual activity possessed by the mutant.

Earlier studies involving *P. gingivalis* SerB653 and HIGK cells demonstrated that SerB653 is an invasin [16], [32], [33]. Since MtSerB2 has a similar sequence architecture compared to SerB653, we looked for rearrangements in human THP-1 cells in the presence of exogenously added MtSerB2 protein and mutants. THP-1 cells can be differentiated into macrophage-like cells and is relevant in the context of *M. tuberculosis*. Human THP-1 cells were incubated with exogenously added full-length protein and mutants *viz* MtSerB2, D341N mutant, ACT domains and the PSP domain alone respectively. The D341N mutant has only residual phosphatase activity as compare to wild type MtSerB2. The ACT and PSP domains were used to find out which domain of MtSerB2 exhibits maximal microtubule re-arrangement activity. Fluorescent microscopy revealed that after 30 minutes of incubation with MtSerB2, THP-1 cells show striking rearrangement of microtubules on the cell surface compared to control cells (**Fig. 8**). This effect was found to have increased at the 2 hour time point. On the other hand D341N, on incubation with THP-1 cells, exhibits minor microtubule rearrangement, presumably because of residual activity of the mutant. The ACT-domains also exhibit almost no re-arrangement of the microtubules. When the PSP domain alone was added, the rearrangement of the microtubules was significant, although the effect was slightly less than that observed with full- length MtSerB2. It also suggests that the protein has new functions in addition to the classic phosphoserine phosphatase activity. The experiments unambiguously demonstrate that the PSP domain and phosphatase activity are important for the observed effects.

## Discussion

MtSerB2 represents an uncharacterized member of the mycobacterial HAD-family phosphatases. The presence of ACT-domains in addition to the classic PSP domain in sequence analysis suggested that MtSerB2 harbors novel functions. The *in vivo* characterization of a *P. gingivalis* invasin, SerB653, has been reported earlier [16]. The studies revealed SerB653 to be important for invasion into host cells and showed how the formerly metabolic enzyme has been adapted by the pathogen as an invasin to facilitate entry into human cells. The *P. gingivalis* SerB653 allelic replacement mutant was further demonstrated to be deficient in internalization and persistence in gingival epithelial cells. The present work demonstrating microtubule rearrangements to THP-1 cells in the presence of exogenously added MtSerB2 are in line with the earlier experiments in *P. gingivalis*, although more experiments have to be performed to demonstrate this fully. Additionally, they go beyond the earlier experiments and demonstrate that the elicited effects depend on the phosphatase activity and also are more localised in the PSP domain. The activity assays show that MtSerB2 is a HAD-family phosphatase, and demonstrates specificity for l-phosphoserine in contrast to other characterized classical phosphoserine phosphatases. In this context, it is interesting that a gram-negative periodontal pathogen and *M. tuberculosis* apparently possess invasins with similar properties.

Another important result is the identification of a specific oligomeric transition in the presence of l- serine. A comparison with the *M. avium* SerB crystal structure (PDB: 3P96) shows that the ACT domains exhibit extensive interactions in the dimer and in fact take part in domain-swapping (**Fig. 1B**). Consequently, binding of ligands to the domains could conceivably alter oligomeric association through changes in their relative spatial disposition. This would presumably be necessary for the protein to transit to other oligomeric states. The functional relevance of this transition can be hypothesized: Since PSPase activity is necessary for the observed microtubule rearrangements, binding to l-serine can act as a feedback regulatory handle for its functions as it is a good inhibitor.

The binding of amino acids/ligands to the ACT domains could elicit an increase/decrease/no effect on MtSerB2 activity. A striking observation in this context is that the observed effects (**Table 4**) are not dependent on the amino acid type. While positively charged Lys exhibits ∼40% activation, Arg acts as a weak inhibitor. Again, while the aromatic acids Tyr and Phe act as activators with more than 30% activation, Trp exhibits inhibitory activity. Consistent with the above observations, the activity of the enzyme is modulated by the ACT domains. In fact, the reduction in the activity of the PSP domain can be mitigated to a large extent by the addition of the ACT domain in 1∶1 stoichiometry.

The results also raise the exciting possibility of designing inhibitors with therapeutic potential that bind to the ACT domains. Dihydroquinolin-4-one derivatives were identified as specific inhibitors of *P. gingivalis* SerB653 that bind to its PSP domain [34]. These were also active at nanomolar levels against *P. gingivalis* growth. Analogously, targeting MtSerB2 may prove to be beneficial in the quest for identifying anti-TB therapeutics with new modes of action. As an initial step, we tested several known phosphatase inhibitors. Among known phosphatase inhibitors, Chlorpromazine hydrochloride exhibits inhibition at nanomolar concentrations. The inhibition by Chorpromazine is non-competitive as also reported earlier in the cases of the classic phosphoserine phosphatases. This agrees with the fact that the compound binds mainly to the PSP domain in contrast to amino acids like l-serine that has highly reduced efficacy against the latter domain. On the other hand, the present results demonstrate the possibility of designing compounds that bind to the ACT domains as potent inhibitors.

Interesting questions involving the molecular mechanisms are thrown up by the present work. An important question is ‘How exactly does the binding of amino acids to the ACT domains get translated to the modulation of enzyme activity?’ We need to distinguish between large-scale conformational changes that can take place between the domains upon binding of the co-factor and smaller changes in conformation that are transmitted to the catalytic domain to presumably prevent a substrate access/reaction from proceeding further, *eg*. in case of inhibition. The present results support a model involving smaller scale conformational changes. The ACT domains of MtSerB2 that are largely involved in amino acid binding (higher affinity for the l-serine); presumably modulate/transmit that information at the molecular level to the catalytic domain. These questions are presently being tested through structural biology approaches.

*Note added in revision*: While this manuscript was being reviewed, Arora et al. (*J. Biol. Chem*, **289**: 25149–25165, 2014) published the characterization of the MtSerB2 enzyme and also reported the identification of its specific inhibitors through high throughput screening. The present enzyme characterization results are broadly in agreement with the above paper.

## Supporting Information

S1 Figure **Sequence & structural alignment of ACT domains.** **(A)** Sequence alignment of respective ACT domains 1 & 2 of Mt SerB2 with Mt PDGH (SerA1) and *E. coli* AHAS ACT domains. **(B)** Structural alignment of the MtSerB2 ACT domains with the crystal structure of Mt_PDGH - l- serine complex (PDB code: 1YGY) and *E. coli* AHAS (PDB code: 2F1F) respectively. The close-up of the l- serine binding site clearly shows the respective structurally conserved Gly residues.
(TIF)

S2 Figure **Inhibition of MtSerB2 by l-serine.** Reaction mix containing protein and l-serine was incubated at 37°C for 30 min and reactions were started by addition of l-phosphoserine. The reactions were incubated again for 30 min at 37°C and inorganic phosphate released was measured by malachite green reagent. Relative activity was plotted against l-serine concentration. The reactions were carried out in triplicate and repeated several times with different batches of purified protein.
(TIF)

S3 Figure **Calibration curve of the Superdex S-200 column.** (GE Biosciences) used in the experiments. A Superdex S-200 column (GE Biosciences), calibrated with low and high molecular weight range markers, was mounted on an AKTA-FPLC system (GEBiosciences) for the experiments. Standard known proteins such as Ovalbumin, Albumin, Conalbumin, Ferritin and Thyroglobulin were used to calibrate the column.
(TIF)
