# Supplementary figures and images for: Characterization of M. tuberculosis SerB2, an Essential HAD-Family Phosphatase, Reveals Novel Properties
Source: PLoS One. 2014 Dec 18;9(12):e115409. doi: 10.1371/journal.pone.0115409 (PMC4270767; doi:10.1371/journal.pone.0115409)

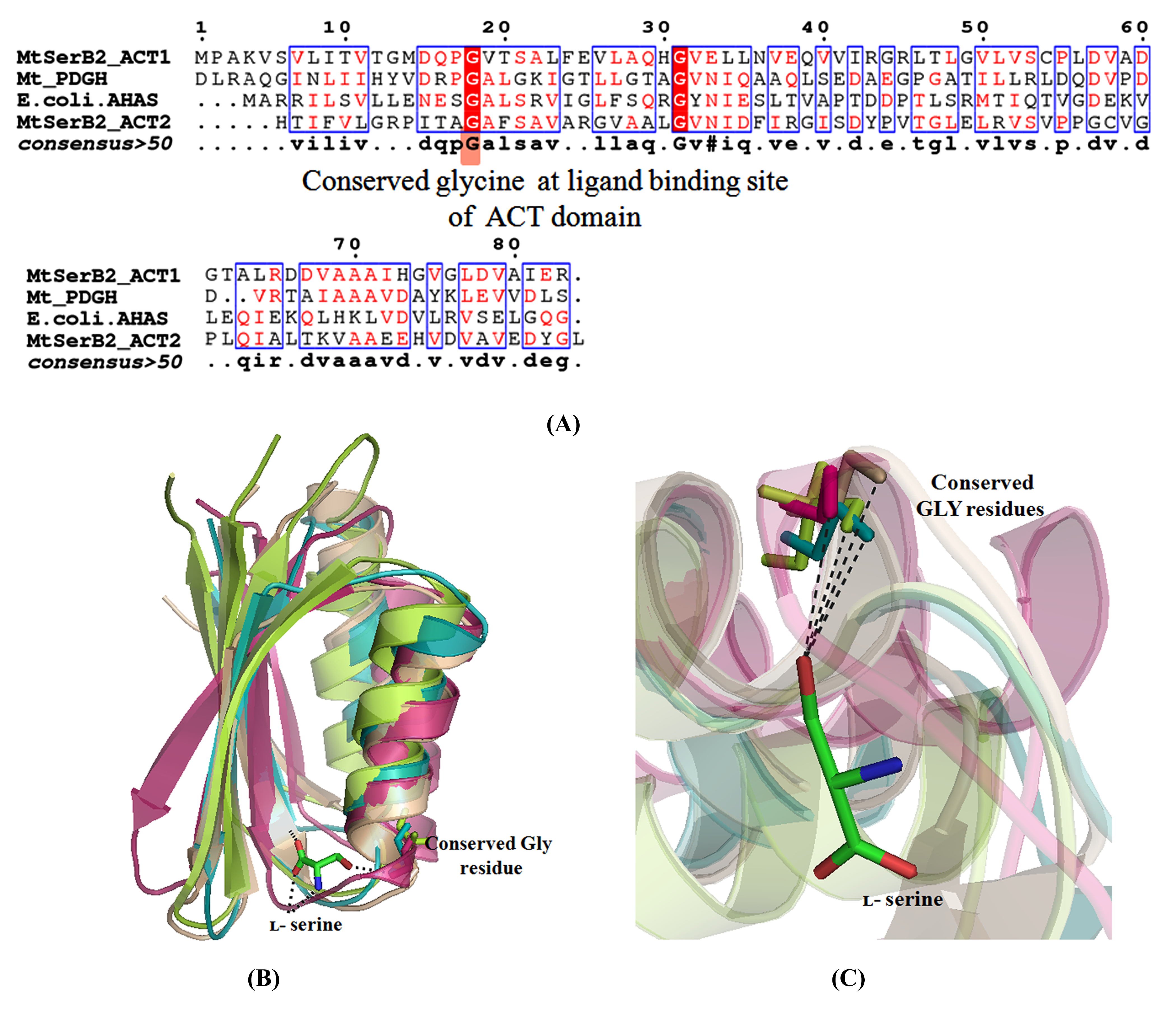

Supplement: S1 Figure — Sequence & structural alignment of ACT domains. (A) Sequence alignment of respective ACT domains 1 & 2 of Mt SerB2 with Mt PDGH (SerA1) and E. coli AHAS ACT domains. (B) Structural alignment of the MtSerB2 ACT domains with the crystal structure of Mt_PDGH - l- serine complex (PDB code: 1YGY) and E. coli AHAS (PDB code: 2F1F) respectively. The close-up of the l- serine binding site clearly shows the respective structurally conserved Gly residues. (TIF) [file pone.0115409.s001.tif]

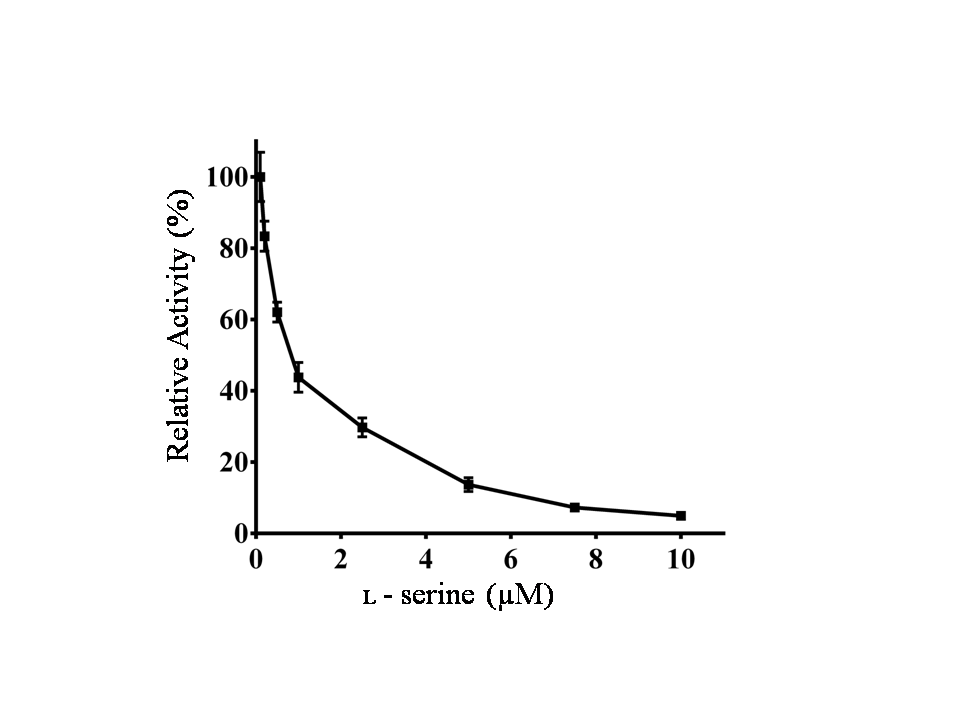

Supplement: S2 Figure — Inhibition of MtSerB2 by l-serine. Reaction mix containing protein and l-serine was incubated at 37°C for 30 min and reactions were started by addition of l-phosphoserine. The reactions were incubated again for 30 min at 37°C and inorganic phosphate released was measured by malachite green reagent. Relative activity was plotted against l-serine concentration. The reactions were carried out in triplicate and repeated several times with different batches of purified protein. (TIF) [file pone.0115409.s002.tif]

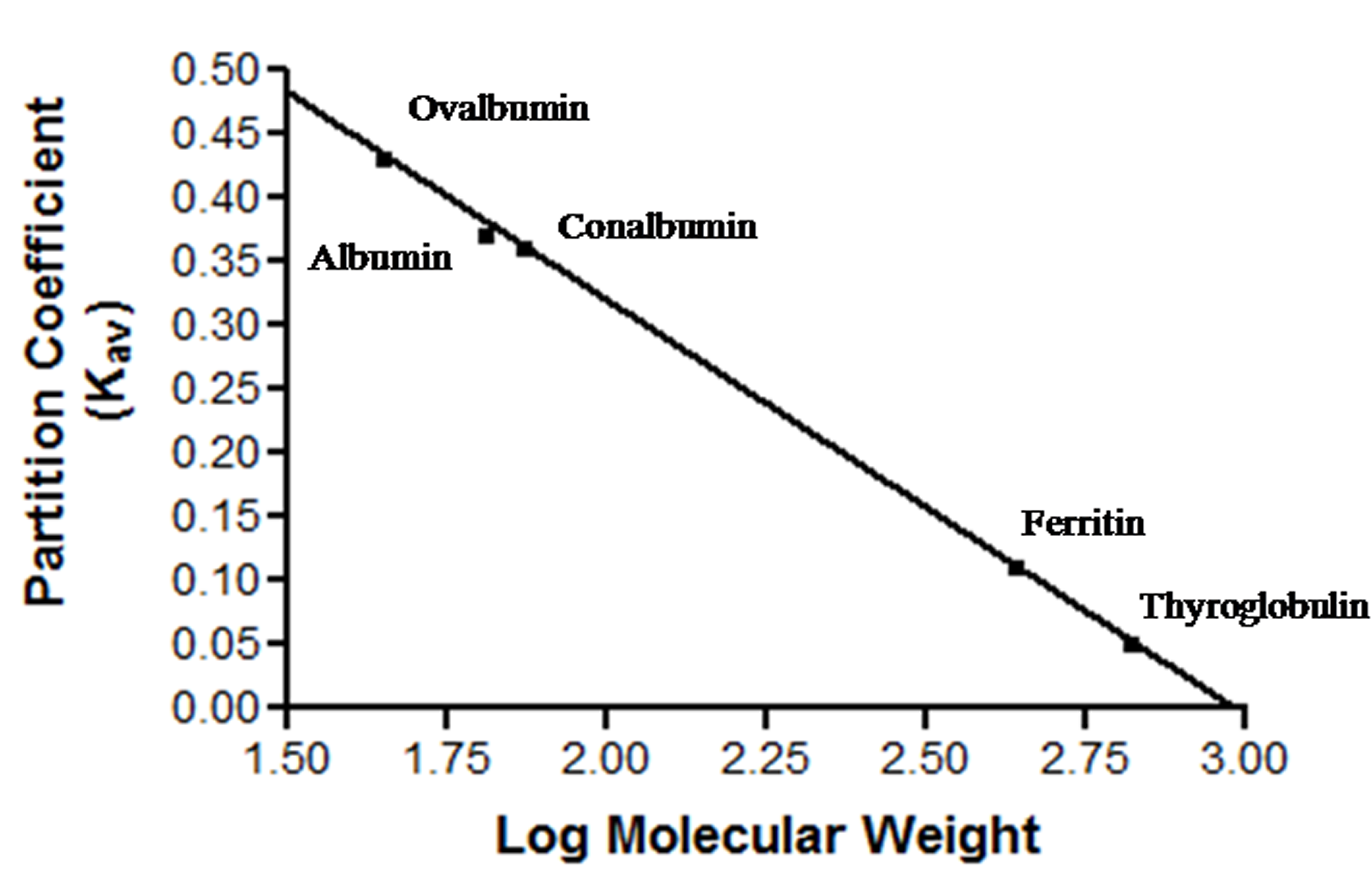

Supplement: S3 Figure — Calibration curve of the Superdex S-200 column. (GE Biosciences) used in the experiments. A Superdex S-200 column (GE Biosciences), calibrated with low and high molecular weight range markers, was mounted on an AKTA-FPLC system (GEBiosciences) for the experiments. Standard known proteins such as Ovalbumin, Albumin, Conalbumin, Ferritin and Thyroglobulin were used to calibrate the column. (TIF) [file pone.0115409.s003.tif]
